# Elevated on-treatment levels of serum IFN-gamma is associated with treatment failure of peginterferon plus ribavirin therapy for chronic hepatitis C

**DOI:** 10.1038/srep22995

**Published:** 2016-03-11

**Authors:** Ming-Ying Lu, Ching-I Huang, Chia-Yen Dai, Shu-Chi Wang, Ming-Yen Hsieh, Meng-Hsuan Hsieh, Po-Cheng Liang, Yi-Hung Lin, Nai-Jen Hou, Ming-Lun Yeh, Chung-Feng Huang, Zu-Yau Lin, Shinn-Cherng Chen, Jee-Fu Huang, Wan-Long Chuang, Ming-Lung Yu

**Affiliations:** 1 Hepatobiliary Division, Department of Internal Medicine and Hepatitis Center, Kaohsiung Medical University Hospital, Kaohsiung, Taiwan; 2 Faculty of Internal Medicine, College of Medicine, and Graduate Institute of Clinical Medicine, and Center for Infectious Disease and Cancer Research, Kaohsiung Medical University, Kaohsiung, Taiwan; 3 Institute of Biomedical Sciences, National Sun Yat-Sen University, Kaohsiung, Taiwan; 4 Department of Internal Medicine, Kaohsiung Municipal Hsiao-Kang Hospital, Kaohsiung Medical University, Kaohsiung, Taiwan; 5 Department of Occupational Medicine, Kaohsiung Municipal Ta-Tung Hospital, Kaohsiung Medical University, Kaohsiung, Taiwan; 6 Department of Preventive Medicine, Kaohsiung Medical University Hospital, Kaohsiung, Taiwan; 7 Graduate Institute of Medicine, College of Medicine, Kaohsiung Medical University, Kaohsiung, Taiwan

## Abstract

Chronic hepatitis C virus (HCV) infection had been associated with cytokine imbalance. Cytokine dynamics in response to peginterferon/ribavirin therapy have an impact on the treatment efficacy for HCV patients. Ninety-two treatment-naive chronic hepatitis C patients were treated with 24 or 48 weeks of peginterferon/ribavirin therapy according to their viral genotypes. Sustained virologic response (SVR) is defined as undetectable HCV RNA throughout a 24-week post-treatment follow-up period. Dynamic serum levels of the following cytokines: (1) Th1-mediated cytokines: IFN-γ, interleukin-2, and TNF-alpha; (2)Th2-mediated cytokines: interleukin-4, interleukin-5, interleukin-6, and interleukin-10 and (3)immuno-modulatory cytokines: interleukin-1β, interleukin-8, and interleukin-12 were determined by Fluorescent Bead immunoassay. Serial dynamic cytokine expression demonstrated that not only elevated IFN-γ concentrations at specific time points but also the total IFN-γ amount was strongly linked to non-response in peginterferon/ribavirin therapy. IFN-γ levels could serve as an independent predictor for SVR analyzed by multivariate logistic regression test. The accuracy of discriminating responders from non-responders was acceptable when IFN-γ cut-off levels were set at 180, 120, and 40 pg/ml at the 4th week, 12th week, and end-of-treatment of therapy, respectively. Elevated on-treatment IFN-γ concentration was significantly associated with treatment failure among interleukin-28B rs8099917TT carriers and those patients failed to achieve rapid virologic response.

Hepatitis C (HCV) is a global problem affecting more than 170 million people worldwide^1^, and approximately 70% of HCV patients will develop chronic hepatitis C^2^. HCV is a flavivirus with a plus-strand RNA genome that exists as at least 6 genotypes (1 to 6) and 50 subtypes^3^. Currently, there is no vaccine to prevent HCV infections. Combination therapy with peginterferon (PegIFN) and ribavirin (RBV) had been the backbone in the treatment for chronic hepatitis C before the IFN-free regimens with direct acting antiviral agents (DAA) available in 2014^4^,^5^,^6^. In the treatment naïve individuals, PegIFN/RBV achieves 40–50% sustained virologic response (SVR) rates in patients infected with in HCV genotype-1 (HCV-1) and 75% in those with HCV-2/3 infections in western countries^7^. Twenty-four weeks of PegIFN/RBV can achieve an SVR rate of 50% and 85% for HCV-1 and HCV-2 Asian patients, respectively^8^,^9^,^10^. The emergence of direct acting antiviral agents, such as telaprevir and boceprevir, has improved the SVR rates substantially and become the regimen of abbreviated therapy for patients infected with HCV-1^11^,^12^,^13^,^14^,^15^. However, the high costs of DAAs limit its clinical application and the PegIFN/RBV regimen remains a vital component of HCV therapy. Therefore, we are interested in surveying some of the predisposing factors associated with achieving SVRs under PegIFN/RBV therapy.

Many viral and host factors contribute to the efficiency of interferon-based antiviral therapy. Rapid virologic response (RVR)^16^, HCV genotypes^17^, viral load^18^,^19^, IL-28B polymorphisms^20^,^21^,^22^,^23^,^24^,^25^, and host microRNAs^26^,^27^ are major predictors for treatment outcomes of PegIFN/RBV therapy. Over 100 different inflammatory cytokines have been identified, which regulate the balance between humoral and cell-mediated immunity^28^. Inflammatory cytokines participate in the defense against viral replications and modulating the host immune function^29^. Th1 cytokines (e.g., interferon-γ and IL-2) are key mediators for host antiviral immunity, while Th2 cytokines (e.g., IL-4 and IL-10) may attenuate these inflammation responses^30^. Interferon and ribavirin have both antiviral and immunomodulatory functions to coordinate the balance between Th1 and Th2 cytokines^31^,^32^. Increased Th2 cytokine production is associated with non-virological response for PegIFN/RBV therapy in chronic HCV patients^33^,^34^. Analysis of the inflammatory cytokines expression during HCV infection could help us realize the mechanism of viral-host interactions and identify the predictors of treatment outcome^35^,^36^,^37^.

We hypothesized that the inflammatory cytokines plays a crucial role in the PegIFN/RBV treatment efficacy of chronic hepatitis C. Most previous clinical studies have analyzed the association between baseline cytokine levels and the outcomes of antiviral therapy. Others have explored the dynamic cytokine expression under interferon stimulation *in vitro*. Because the expression of inflammatory cytokines is consecutive and fluctuating during the anti-viral course, our study provides a more comprehensive viewpoint. We conducted a clinical study to survey the dynamics of inflammatory cytokines during PegIFN/RBV therapy for HCV. This study was aimed to investigate the relationship between on-treatment cytokine variations and treatment efficacy. We tried to identify immunological factors to predict the outcomes of antiviral therapy.

## Materials and Methods

### Subjects

Ninety-two chronic hepatitis C patients who treated with PegIFN/RBV combination therapy were enrolled in the study. The inclusion criteria in this study were as follows:(a)adults aged more than 18 years old with the presence of anti-HCV antibodies and detectable serum HCV RNA for more than 6 months and (b) serum Alanine aminotransferase (ATL) more than 1.5-fold the normal range. Patients were excluded if they had the following concurrent diseases or conditions: (1) co-infection with hepatitis B, hepatitis D, or human immunodeficiency virus, (2)decompensated liver cirrhosis, (3) overt hepatic failure, (4) renal function impairment (eGFR < 50 ml/min), (5) primary biliary cirrhosis, (6) autoimmune hepatitis, (7) Wilson disease, (8) sclerosing cholangitis, (9) α_1_-antitrypsin deficiency, (10) preexisting psychiatric disorder, (11) current or past history of alcohol abuse (≧20 g daily), (12) liver transplantation, or (13) the presence of hepatocellular carcinoma or other malignancy. All of the patients received liver biopsies to prove the severity of the chronic hepatitis while enrolled in this study. Liver histology was graded and staged on the basis of the scoring system proposed by Knodell and Scheuer^38^,^39^. A single pathologist was blinded to make the diagnosis for each sample. Written informed consent was obtained from each participant, and the study design in concordance with ethical guidelines was approved by the Ethics Committee of Kaohsiung Medical University Hospital.

### Combination PegIFN and ribavirin therapy

All of the participants subcutaneously received peg-interferon α-2a (180 μg/week) plus weight-based ribavirin (1000 mg/day for weights <75 kg or 1200 mg/day for weights >75 kg). A 24-week regimen was administered to patients with HCV-2/3 or HCV-1 with rapid virologic response (RVR). For those with HCV-1 who failed to achieve RVR, the treatment course was extended to 48 weeks. RVR was defined as negative for HCV RNA after 4 weeks of treatment. Sustained virologic response (SVR) was defined as clearance of the serum HCV RNA at the end of the therapy and lasting for more than 24 weeks after the cessation of therapy. Patients failed to achieve SVR were classified as non-sustained virologic response (non-SVR).

### Quantification of HCV RNA and genotyping

HCV antibodies (anti-HCV) were detected by using a third-generation, commercialized enzyme-linked immunosorbent assay kit (Abbott Laboratories, Chicago, IL, USA). HCV RNA was quantified by a real-time polymerase chain reaction assay^40^ (detection limit: 50 IU/ml; RealTime HCV; Abbott Molecular, Des Plaines IL, USA). HCV genotypes were identified by the method proposed by Okamoto *et al*.^41^.

### SNP genotyping

*The IL28B* rs8099917 genotype is significantly associated with the treatment outcome of PegIFN/RBV therapy and has been proven by genome-wide association studies and validation studies in Asian cohorts^21^,^42^,^43^. Genotypes were identified by the ABI TaqMan® SNP genotyping assays (Applied Biosystems, CA, USA) using the pre-designed commercial genotyping probe (ABI Assay ID: C_11710096_10) according to the manufacturer’s recommendations.

### Cytokine measurements

A Fluorescent Bead immunoassay (Bio-Rad Laboratories, Hercules, CA, USA) was used to measure the serum cytokine levels according to the manufacturer’s recommendations. Cytokine concentrations were calculated by using a reference standard curve made with various concentrations of the standards. Each sample was tested in triplicate and the average was calculated. Serum samples were collected from the participants at the baseline; 2nd, 4th, and 12^th^ week of treatment; end-of-treatment (EOT); and three-month follow-up. The following cytokines were analyzed: (1)Th1-mediated cytokines: IFN-γ (interferon-gamma), IL-2 (interleukin-2), and TNF-α (tumor necrosis factor-alpha);(2)Th2-mediated cytokines: IL-4 (interleukin-4 ), IL-5 (interleukin-5), IL-6 (interleukin-6), and IL-10 (interleukin-10); and (3)immuno-modulatory cytokines: IL-1β (interleukin-1β), IL-8 (interleukin-8), and IL-12 (interleukin-12).

### Statistics

The Student’s t test was used to analyze the continuous variables. The Chi-square (*X*^2^)-test or Fisher’s exact test was used to assess the categorical variables. The multivariate logistic regression test was further performed to identify the independent factors to predict the SVR. The area under the curve (AUC) was calculated using receiver-operating characteristics (ROC) analysis. The optimum cut-off value of serum cytokine concentration to divide the risk strata was calculated by the Yauden index. The mutual effects of time and cytokine levels between the SVR and non-SVR groups were analyzed by repeated measure ANOVA. A log_10_-based transformation of the cytokine levels improved the normal distributions. A two-tailed *p*-value < 0.05 was considered statistically significant. All of the statistical analyses were performed using the Statistic Packages for Social Science Program (SPSS version 13.0 for windows, SPSS Inc., Chicago, IL, USA).

## Results

### Subjects

The demographic characteristics of the study subjects are shown in Table 1. Of the 92 HCV patients, 73 (79.3%) patients achieved SVR. Forty-four patients (47.8%) had HCV-1b infection. Sixty-two patients (82.7%) carried the IL28B rs8099917 TT genotype and 13 patients (17.3%) carried the unfavorable TG/GG genotype. All of the baseline features were similar between the SVR and non-SVR groups except for the baseline HCV viral load and the GPT. There were significantly more patients with a high viral load (>4 × 10^5^ IU/ml) in the non-SVR group than in the SVR group (66.7% vs. 34.2%, p = 0.012). The GPT level was significantly higher in the SVR group than in the non-SVR group (mean ± SD of 164.5 ± 107.1 vs. 119.3 ± 71.3 IU/L, p = 0.034).

### Serial serum cytokine expression levels between the SVR and non-SVR groups

A total of ten cytokines were examined in this study, including three Th1-mediated cytokines (IFN-γ, IL-2, and TNF-α), four Th2-mediated cytokines (IL-4, IL-5, IL-6, and IL-10), and three immuno-modulatory cytokines (IL-1β, IL-8, and IL-12). We found that the baseline levels of IL-2 and IL-4 were significantly elevated in the non-SVR group compared with the SVR group (mean ± SE of 86.9 ± 13.8 vs. 52.7 ± 6.2 pg/ml, p = 0.017 for IL-2 and 649.1 ± 139.1 vs. 363.1 ± 61.3 pg/ml, p = 0.040 for IL-4) (Supplementary Table 1). However, both of the groups could not achieve statistical significance after the multivariate adjustment (adjusted OR = 1.01, 95% C.I = 1.00~1.02, p = 0.059 for IL-2 and adjusted OR = 1.00, 95% C.I = 1.00~1.00, p = 0.068 for IL-4).

**Figure 1:**
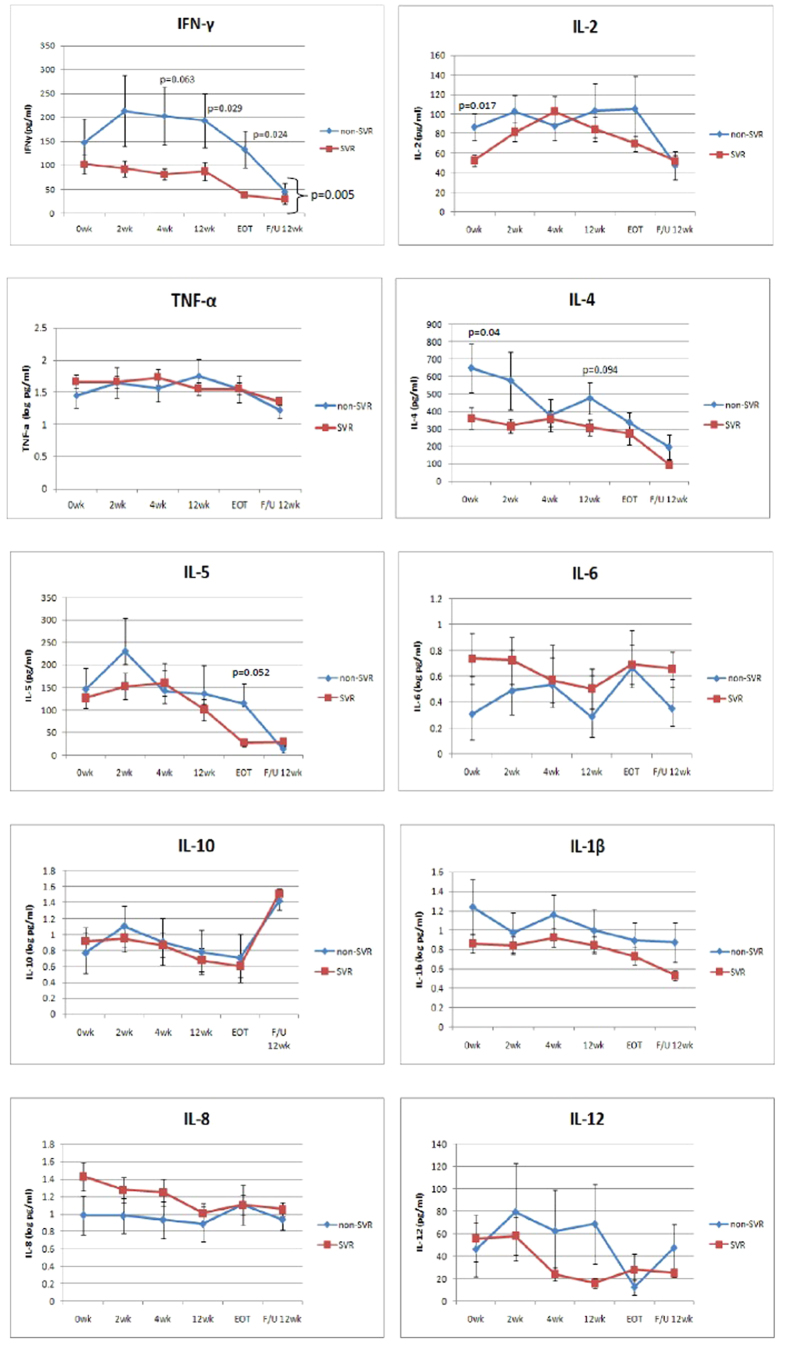
Serial serum cytokine expression levels between SVR and non-SVR. Th1-mediated cytokine: IFN-γ, IL-2, TNF-α. Th2-mediated cytokine: IL-4, IL-5, IL-6, IL-10. Immuno-modulatory cytokine: IL-1β, IL-8, IL-12. p.s. SVR n = 73, non-SVR n = 19; EOT: end of treatment.

Serial serum levels of the ten cytokines during and after PegIFN/RBV therapy between the SVR and non-SVR groups are shown in Fig. 1. We found that only serial dynamics of IFN-γ revealed a marked difference between the SVR and non-SVR groups (p = 0.005) by repeated measure ANOVA on the mutual effects of time and the ten cytokines analyzed. The serum levels of IFN-γ were significantly elevated in the non-SVR group compared with the SVR group at 4th and 12th week of treatment and the end-of-treatment. (mean ± SE of 203.4 ± 60.2 vs. 81.6 ± 11.6 pg/ml, p = 0.063 at the 4th week; 193.8 ± 57.4 vs. 88.0 ± 19.2 pg/ml, p = 0.029 at the 12th week; and 133.6 ± 38.5 vs. 38.1 ± 6.2 pg/ml, p = 0.024 at the end-of-treatment, respectively). (Figure 1) We therefore chose IFN-γ for further analysis.

### The association between IFN-γ and SVR

To evaluate the impact of IFN-γ concentration on the response to PegIFN/RBV therapy, we divided the subjects into high or low IFN-γ groups. By performing an ROC analysis, we established cut-off values of 180, 120, and 40 pg/ml of IFN-γ at the 4th and 12th weeks of therapy and end-of-treatment, respectively. At the 4th week of PegIFN/RBV therapy, patients with lower IFN-γ concentrations (<180 pg/ml) had greater SVR rates than those with higher IFN-γ levels. (86.5% vs. 50.0%, p = 6.0 × 10^−4^). Multivariate analysis revealed that IFN-γ levels at week 4 were an independent factor for predicting SVR (adjusted OR = 6.79, 95% C.I = 1.91~24.09, p = 3.0 × 10^−3^). Similar results were detected at the 12th week and end-of-treatment for PegIFN/RBV therapy. The SVR rate was significantly higher in low IFN-γ groups (<120 pg/ml at the 12th week or <40 pg/ml at the end-of-treatment) compared with the high IFN-γ groups (89.8% vs. 60.6%, p = 9.0 × 10^−4^ at the 12th week and 89.7% vs. 61.8%, p = 1.4 × 10^−3^ at the end-of-treatment). After the multivariate adjustment, the high IFN-γ groups had a significantly higher risk to fail to achieve SVR (adjusted OR = 11.55, 95% C.I = 3.05~43.77, p = 3.2 × 10^−4^at the 12th week and adjusted OR = 4.93, 95% C.I = 1.52~15.93, p = 7.7 × 10^−3^ at the end-of-treatment) (Table 2).

**Figure 2:**
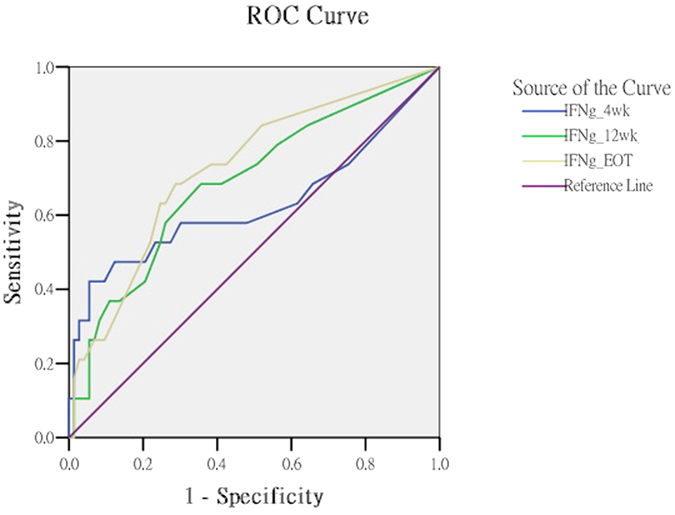
ROC curve analysis.

When we set up the IFN-γ cut-off level of 180 pg/ml at the 4th week, the area under the ROC curve (AUC) was 0.627 (95% C.I = 0.456~0.800, p = 0.086). When the cut-off values were set up as 120 pg/ml at the 12th week and 40 pg/ml at the end-of-treatment, the AUC had significantly improved to 0.689 (95% C.I = 0.550~0.827, p = 0.012) and 0.726 (95% C.I = 0.596~0.855, p = 0.003), respectively (Table 3 and Fig. 2).

### Subgroup analysis (high vs. low viral load)

Because HCV viral load, HCV genotype, IL-28B genotype, and RVR were major predictors for SVR, we further stratified these patients according to presence of above factors. Among the patients with a high viral load (>4 × 10^5^ IU/ml), low IFN-γ groups at both the 4th and 12th week had a significantly higher SVR rate than the high IFN-γ groups (80.8% vs. 36.4%, OR = 2.22, 95% C.I = 0.99~4.96, p = 0.018 at the 4th week and 84.2% vs. 50.0%, OR = 1.68, 95% C.I = 1.02~2.78, p = 0.038 at the 12th week, respectively). However, this effect did not achieve statistical significance at the end-of-treatment with the PegIFN/RBV therapy. Among those with a low viral load (≦4 × 10^5^ IU/ml), low IFN-γ groups at both the 12th week and the end-of-treatment achieved a significantly higher SVR rate compared with the high IFN-γ groups (94.9% vs. 73.3%, OR = 1.29, 95% C.I = 0.95~1.77, p = 0.044 at the 12th week and 94.9% vs. 73.3%, OR = 1.29, 95% C.I = 0.95~1.77, p = 0.044 at the end-of-treatment). Additionally, there were no differences between the responder and non-responder IFN-γ levels at the 4th week (Table 4).

### Subgroup analysis (HCV genotype 1b vs. non-1b)

Because HCV genotype 1b had an unfavorable treatment outcome, we stratified patients into genotype 1b or non-1b. Among the patients with the HCV 1b genotype, the SVR rate was significantly higher in the low IFN-γ groups compared with the high IFN-γ groups at both the 4^th^ week and the end-of-treatment (84.2% vs. 16.7%, OR = 5.05, 95% CI = 0.84~30.40, p = 0.002 at the 4th week and 88.9% vs. 52.9%, OR = 1.68, 95% C.I = 1.05~2.68, p = 0.012 at the end-of-treatment, respectively). At the 12th week, the IFN-γ level (120 pg/ml) only had borderline statistical significance to predict the SVR (85.7% vs. 56.3%, OR = 1.54, 95% C.I = 0.96~2.41, p = 0.067). Among the patients with the HCV non-1b genotype, the high IFN-γ group had a significantly higher risk to fail to achieve SVR compared with the low IFN-γ group at the 12th week (93.5% vs. 64.7%, OR = 1.45, 95% C.I = 1.01~2.08, p = 0.017). Neither of the subgroups achieved statistical significance at the 4th week or the end-of-treatment (88.9% vs. 66.7%, OR = 1.33, 95% C.I = 0.88~2.02, p = 0.094 at the 4th week and 90.3% vs. 70.6%, OR = 1.28, 95% C.I = 0.92~1.78 at the end-of-treatment, p = 0.112, respectively) (Table 5).

### Subgroup analysis (IL28B TT vs. GT genotype)

In the previous study, we found that IL28B rs8099917 TT carriers were favorable to achieve SVR^21^,^42^. We further stratified patients according to the IL28B rs8099917 genotypes. Among the patients with the IL28B rs8099917 TT genotype, the low IFN-γ groups had a significantly higher SVR rate than the high IFN-γ groups at 12th week and at the end-of-treatment (90.7% vs. 63.2%, OR = 1.44, 95% C.I = 1.01~2.05, p = 0.026 at the 12th week and 90.7%vs. 63.2%, OR = 1.44, 95% C.I = 1.01~2.05, p = 0.026 at the end-of-treatment, respectively). At the 4th week of treatment, the SVR rate differences between these subgroups were borderline statistically significantly (86.3% vs. 63.6%, OR = 1.36, 95% C.I = 0.86~2.15, p = 0.094). Among the patients with the IL28Brs8099917 GT genotype, there were no SVR rate differences between the subgroups at any of the time points due to the small sample size (Table 6).

### Subgroup analysis (RVR vs. Non-RVR)

Because RVR is the single best predictor for SVR^44^, we further stratified the patients into RVR and non-RVR subgroups. We found that IFN-γ remained an important predictor for SVR in patients with non-RVR, but was not important for those patients with RVR. The high IFN-γ group had a significantly higher risk to fail to achieve SVR compared with the low IFN-γ group (80.0% vs. 38.5%, OR = 3.08, 95% C.I = 0.86~11.03, p = 0.06 at the 4th week and 70.6% vs. 31.6%, OR = 2.33, 95% C.I = 1.05~5.16, p = 0.019 at both the 12th week and the end-of-treatment). Because almost all of the patients with RVR could eventually achieve SVR (98.2%), the effect of IFN-γ was obscured in the RVR subgroup (Table 7).

### Area under the serial IFN-γ expression curve

**Figure 3:**
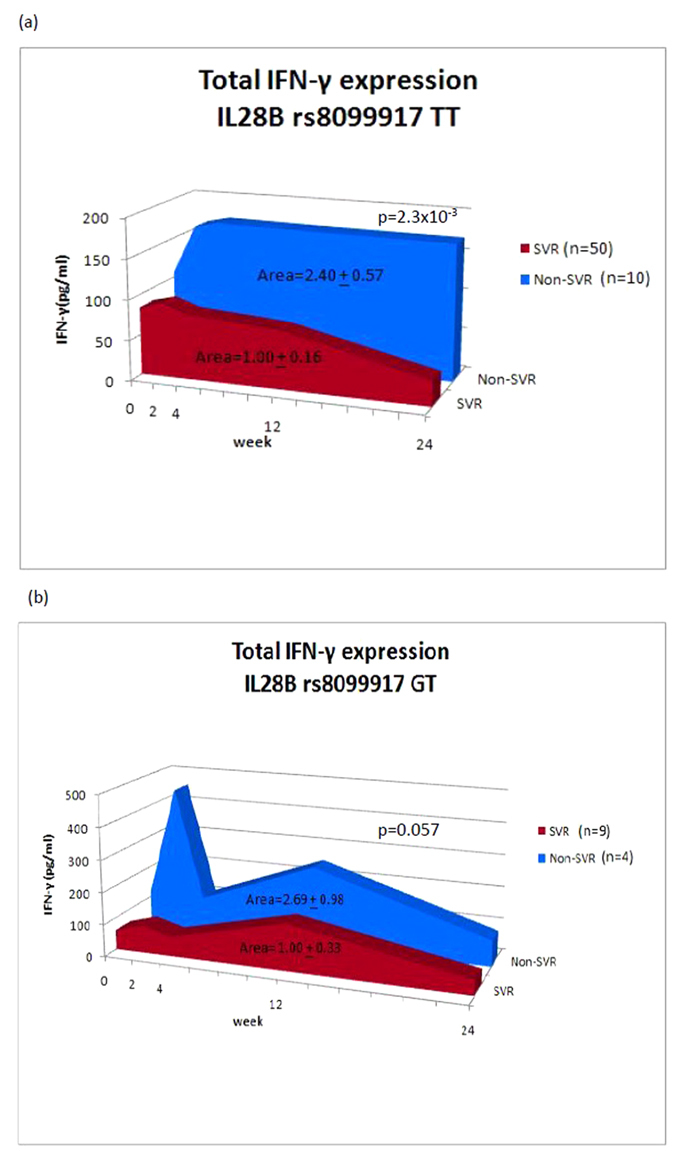
Area under the serial IFN-γ expression curve during PegIFN/RBV therapy.

Because cytokine expression is continuously changing during PegIFN/RBV treatment, we calculated the area under the serial IFN-γ expression curve to stand for the total IFN-γ amount during the treatment course. Among the IL28B rs8099917 TT carriers, the total amount of IFN-γ significantly elevated (2.40-fold) in the non-SVR group compared with the SVR group. (Area = 2.40 ± 0.57 in the non-SVR group vs. 1.00 ± 0.16 in the SVR group, p = 2.3 × 10^−3^)(Fig. 3a). For those carrying the unfavorable GT genotype, the total amount of IFN-γ in the non-SVR group was borderline greater than the SVR group (area = 2.69 ± 0.98 in the non-SVR group vs. 1.00 ± 0.33 in the SVR group, p = 0.057) (Fig. 3b).Because either the HCV genotype or the viral load was comparable between these subgroups, the viral factor regarding the induction of IFN-γ expression could be neglected.

## Discussion

This serial cytokine dynamic study demonstrated that IFN-γ could serve as an important biomarker of achieving SVR. Not only the elevated IFN-γ concentrations at specific time points but also the total IFN-γ amounts were strongly linked to non-response with PegIFN/RBV treatment. IFN-γ was an independent predictor for SVR as analyzed by the multivariate logistic regression test. These results were also replicated in the subgroup analyses when patients were further stratified by viral load, HCV genotypes, IL28B rs8099917genotypes, and RVR. This study provided evidence that up-regulation of IFN-γ plays an important role in the poor outcomes of PegIFN/RBV therapy. Although the viral load, HCV genotype, IL28B polymorphisms, and RVR are well-known viral and host factors for predicting SVR, PegIFN/RBV therapies are not effective in all treated patients. Monitoring IFN-γ levels can aid clinicians to further identify high risk patients who may fail PegIFN/RBV therapy and allow for the adoption of appropriate strategies for more personalized medicine.

Both IFN-γ and IL-28B (also designated as IFNL3 or IFN-λ3) belong to the IFN family even though they possess dissimilar biological features. The gene locus of IFN-γ and IL28B are located in chr.12q24.1 and chr.19q13.13, respectively. IL28B binds to IFNLR1 or the IL-10R2 receptor and activates IFN-stimulated gene factors 3 (ISGF3) and STAT1 homodimers. IFN-γ binds to IFNGR1or the IFNGR2 receptor to activate STAT1 and induces a distinct set of interferon-stimulated genes (ISGs). While virtually all cells express IFN-γ receptors, IFN-γ is mainly produced by natural killer (NK) T cells and antigen-specific T cells^45^. The following three distinct NK cell subsets have been identified: (1) CD56^bright^, (2) CD56^dim^, and (3) CD56^neg^CD16^pos^. CD56^bright^cells display less cytotoxicity and have the capacity to produce amounts of abundant cytokines including IFN-γ. By contrast, the CD56^dim^ subset is strongly cytotoxic and characterized by abundant perforin-containing granule contents. CD56^dim^ NK cells produce less cytokines than their CD56^bright^ counterparts. The CD56^neg^CD16^pos^ NK subset seems to be terminally differentiated and has impaired cytotoxicity and less cytokine production^46^. Baseline proportion of CD56^dim^ NK cells was significantly higher in SVR than in non-responder subjects. The change in NK cell phenotypes and functions was independent of IL28B favorable genotypes^47^. We speculate several possibilities to explain the differential expression of IFN-γ between the SVR and non-SVR groups. First, mutations of IFN-γ gene or variations in its gene regulation may result in altered IFN-γ expression. Mutation of IFN-γ receptors may decrease the binding affinity to its agonist and free form IFN-γ will be detected in serum. Moreover, NK cells may be involved in the treatment efficiency of interferon-based therapies by changing the phenotypes of NK cells toward reinforced cytotoxicity and reduced IFN-γ production. Additional studies need to be performed to prove or disprove the above speculations.

Several previous studies reported the association between the IFN-γ gene polymorphisms and treatment response to IFN-based therapy in HCV-infected patients. Huang *et al.* reported a -764G/C single nucleotide polymorphism (SNP) located in proximal IFN-γ promoter region was significantly associated with sustained virologic response. The G allele confers a two- to three-fold higher promoter activity and stronger binding affinity to heat shock transcription factor than the C allele^48^. +874 T/A SNP of IFN-γ gene can directly influence the expression level of IFN-γ^49^, but it failed to link the association between the +874T/A SNP and response to the combination therapy of high dose interferon and ribavirin^50^. Consistent with current study, Wan at al. reported serum levels of Th1 and Th2 cytokines (IFN-γ, TNF, IL-2, IL-4, IL-5, and IL-10) were higher in non-SVR patients than in SVR patients treated with 24-week interferon-α^51^. These phenomenon indicated that Th1/Th2 cytokine imbalance may be associated with ineffective anti-HCV immune response.

A variety of innate and adaptive immune factors are involved in the progression of chronic HCV infection. Upon HCV infection, the host activates the IFN-mediated immune system to defend virus invasion^52^. IFN can drive the synthesis of more than three hundred IFN-stimulated genes (ISGs) to restrict the HCV replication cycles by triggering the JAK/STAT pathway^53^,^54^. On the contrary, HCV have developed numerous elaborate strategies to escape the surveillance of host immune system^55^. For example, HCV NS3 protease cleaves the mitochondrial antiviral signaling protein (MAVS) adapters, which in turn inhibit interferon production^56^. HCV-mediated phosphorylation of PKR (ds-RNA dependent protein kinase R) suppresses the eukaryotic translation initiation factor (eIF2α) and decreases the ISG protein expression^57^. HCV core protein activates the SOCS3 (suppressor of cytokine signaling 3), a repressor of STAT1, and reduces the IFN-induced ISG expression^58^. Although the IFN-induced innate immune response can suppress HCV replication in the early phase, the clearance of HCV virus was mainly depend on T cell-mediated adaptive immunity in the late phase^59^,^60^. Defective expression of NK receptors contribute to NK cells and CD8 + T cells dysfunction, which lead to the persistence of HCV infection^61^. Genetic variations of HCV genome can prevent the recognition of infected hepatocytes by HCV-specific T cells^62^.

It is interesting that we found that it is difficult for patients with high IFN-γ levels to achieve SVR compared to those with low IFN-γ levels. Host-mediated hepatic inflammation and fibrosis is in part induced by endogenous IFNs in chronic HCV infection^63^. For patients with pre-activated endogenous IFN systems, numerous of ISGs have over-expressed before therapy. The administration of exogenous interferon will not further promote the expression of ISGs^64^. The mechanism of refractoriness of IFNα signaling remains unclear. There is evidence that USP18 (Ubl carboxyl-terminal hydrolase 18) results in the prolonged desensitization of IFNα signal transduction^65^. Therefore, induction of endogenous IFN is ineffective in clearing of virus during chronic HCV infection^52^,^66^. Patients with more robust pre-activated endogenous IFN system and ISGs levels tend to fail to achieve SVR in response to PegIFN/RBV therapy^67^,^68^. Inhibition of viral replication may revert the refractory endogenous IFN system to activated status. Previous study found that suppression of viral replication by miR-122 cause a reduction of ISG expression in the liver^69^. One of the reasons why direct acting anti-viral drugs (DAAs) can achieve the high SVR rate is restoring interferon sensitivity in patients with refractory interferon systems^13^,^70^. The precise mechanisms how HCV interferes with IFN signaling and its interactions with HCV-specific T lymphocytes remains a mystery in the field.

There are several limitations in our study. The extended 48-week PegIFN/RBV therapy for HCV-1 had significantly higher SVR rates (79%) than the 24-week course (59%)^8^. Seventy-eight percent of the HCV-1 patients with RVR could achieve an SVR when 24-week PegIFN/RBV regimen was administered^9^,^71^. The National Health Insurance in Taiwan universally reimbursed a 24-week regimen regardless of HCV genotype before 2011. In this study, 59.1% of HCV-1b patients who failed to achieve RVR could not afford the full 48-week PegIFN/RBV therapy. This may lead to misclassification of some expected SVR subjects into the non-SVR group. The overall SVR rate for HCV-1b patients was up to 75% in this study, which was comparable with our previous reports. If the HCV-1b patients without RVR treated with less than 48weeks of PegIFN/RBV therapy were excluded, the results remained statistically significant (Supplementary Table 3). It is likely that the appropriate IFN-γ cut-off value to predict SVR varies among different races. Moreover, peripheral cytokine expression may not reflect the actual conditions in the liver. The above findings must be cautiously interpreted for patients co-infected with other viruses, inflammatory disease, or malignancy. These findings require more studies to definitively confirm their relevancy.

## Conclusions

This study provides evidence that increased IFN-γ expression is highly linked to poor outcomes in PegIFN/RBV therapy. Monitoring IFN-γ levels can aid clinicians further to identify high risk patients who may fail PegIFN/RBV therapies and enable appropriate decisions to be made for more personalized medicine in these cases.

## Additional Information

**How to cite this article**: Lu, M.-Y. *et al.* Elevated on-treatment levels of serum IFN-gamma is associated with treatment failure of peginterferon plus ribavirin therapy for chronic hepatitis C. *Sci. Rep.* **6**, 22995; doi: 10.1038/srep22995 (2016).

## Supplementary Material

Supplementary Information

## Figures and Tables

**Table 1:** Baseline characteristics of HCV patients.

	SVR	Non-SVR	*p*-vale
n	73	19	
Sex (male, %)	41(56.2%)	8(42.1%)	0.274
Age (mean ± SD)	52.0 ± 8.3	51.7 ± 13.1	0.919
GOT(IU/L) (mean ± SD)	99.5 ± 57.8	90.6 ± 60.1	0.553
GPT(IU/L) (mean ± SD)	164.5 ± 107.1	119.3 ± 71.3	0.034
Fibrosis			
0–2	53(74.6%)	14(73.7%)	0.932
3–4	18(25.4%)	5 (26.3%)	
Viral load			
high (>4 × 10^5^ IU/ml)	25 (34.2%)	12 (66.7%)	0.012
Low (≦4 × 10^5^ IU/ml)	48 (65.8%)	6 (33.3%)	
HCV genotype			
1b	33(45.2%)	11(57.9%)	0.324
Non-1b	40(54.8%)	8(42.1%)	
IL28B_rs8099917			
TT	51(85.0%)	11(73.3%)	0.279
GT	9(15.0%)	4(26.7%)	

**Table 2:** The association between IFN-γ and sustained virologic response in HCV patients.

IFN-γ (pg/ml)	SVR	>Non-SVR	*X*^*2*^ p-value	Multivariate p-value	*p*-value
	(n = 73)	(n = 19)		OR (95% C.I)	
IFN-γ 4wk					
<180	64(86.5%)	10(13.5%)	6.0 × 10^−4^	6.79 (1.91~24.09)	3.0 × 10^−3^
≧180	9(50.0%)	9(50.0%)			
IFN-γ 12wk					
<120	53(89.8%)	6(10.2%)	9.0 × 10^−4^	11.55 (3.05~43.77)	3.2 × 10^−4^
≧120	20(60.6%)	13(39.4%)			
IFN-γ EOT					
<40	52(89.7%)	6(10.3%)	1.4 × 10^−3^	4.93 (1.52~15.93)	7.7 × 10^−3^
≧40	21(61.8%)	13(38.2%)			

p.s. Adjusted for viral load and GPT. EOT: end of treatment.

**Table 3:** Area under the curve of IFN-γ.

Week	Area	Std. Error	p-value	95% C.I
4week	0.627	0.088	0.086	0.456~0.800
12week	0.689	0.071	0.012	0.550~0.827
EOT	0.726	0.066	0.003	0.596~0.855

p.s. EOT: end of treatment.

**Table 4:** The association between IFN-γ and sustained virologic response in HCV subgroups (High vs. Low viral load).

IFN-γ (pg/ml)		Low viral load (≦4 × 10^5^ IU/ml)				High viral load (>4 × 105 IU/ml)		Odds ratio
	SVR	Non-SVR	X^2^ or Fisher’s	Odds ratio	SVR	Non-SVR	X^2^ or Fisher’s	
	(n = 48)	(n = 6)	*p*-value	OR (95% C.I)	(n = 25)	(n = 12)	*p*-value	OR (95% C.I)
IFN-γ 4wk								
<180	43(91.5%)	4(8.5%)	0.168	1.28 (0.80~2.06)	21 (80.8%)	5 (19.2%)	0.018	2.22 (0.99~4.96)
≧180	5(71.4%)	2(28.6%)			4 (36.4%)	7 (63.6%)		
IFN-γ 12wk								
<120	37 (94.9%)	2 (5.1%)	0.044	1.29 (0.95~1.77)	16 (84.2%)	3 (15.8%)	0.038	1.68 (1.02~2.78)
≧120	11 (73.3%)	4 (26.7%)			9 (50.0%)	9 (50.0%)		
IFN-γ EOT								
<40	37 (94.9%)	2 (5.1%)	0.044	1.29 (0.95~1.77)	15 (78.9%)	4 (21.1%)	0.170	1.42 (0.89~2.28)
≧40	11 (73.3%)	4 (26.7%)			10 (55.6%)	8 (44.4%)		

p.s. EOT: end of treatment.

**Table 5:** The association between IFN-γ and sustained virologic response in HCV subgroups (HCV genotype 1b vs. non-1b).

IFN-γ (pg/ml)	HCV genotype Non-1b				HCV genotype 1b			
	SVR	Non-SVR	X^2^ or Fisher’s	Odds ratio	SVR	Non-SVR	X^2^ or Fisher’s	Odds ratio
	(n = 40)	(n = 8)	p-value	OR (95% C.I)	(n = 33)	(n = 11)	p-value	OR (95% C.I)
IFN-γ 4wk								
<180	32 (88.9%)	4 (11.1%)	0.094	1.33 (0.88~2.02)	32 (84.2%)	6 (15.8%)	0.002	5.05 (0.84~30.40)
≧180	8 (66.7%)	4 (33.3%)			1 (16.7%)	5 (83.3%)		
IFN-γ 12wk								
<120	29 (93.5%)	2 (6.5%)	0.017	1.45 (1.01~2.08)	24 (85.7%)	4 (14.3%)	0.067	1.54 (0.96~2.41)
≧120	11 (64.7%)	6 (35.3%)			9 (56.3%)	7 (43.7%)		
IFN-γ EOT								
<40	28(90.3%)	3(9.7%)	0.112	1.28 (0.92~1.78)	24 (88.9%)	3 (11.1%)	0.012	1.68 (1.05~2.68)
≧40	12(70.6%)	5(29.4%)			9 (52.9%)	8 (47.1%)		

p.s. EOT: end of treatment.

**Table 6:** The association between IFN-γ and sustained virologic response in HCV subgroups (IL28B_rs8099917 genotype TT vs. GT).

IFN-γ (pg/ml)	IL28B_rs 8099917 TT				IL28B_rs 8099917 GT			
	SVR	Non-SVR	X^2^ or Fisher’s	Odds ratio	SVR	Non-SVR	X^2^ or Fisher’s	Odds ratio
	(n = 51)	(n = 11)	p-value	OR (95% C.I)	(n = 9)	(n = 4)	p-value	OR (95% C.I)
IFN-γ 4wk								
<180	44 (86.3%)	7 (13.7%)	0.094	1.36(0.86~2.15)	9 (81.8%)	2(18.2%)	0.077	–
≧180	7 (63.6%)	4 (36.4%)			0 (0.0%)	2(100.0%)		
IFN-γ 12wk								
<120	39 (90.7%)	4 (9.30%)	0.026	1.44(1.01~2.05)	5 (83.3%)	1 (16.7%)	0.559	1.46(0.70~3.04)
≧120	12 (63.2%)	7 (36.8%)			4 (57.1%)	3 (42.9%)		
IFN-γ EOT								
<40	39 (90.7%)	4 (9.30%)	0.026	1.44 (1.01~2.05)	6 (85.7%)	1 (14.3%)	0.266	1.71(0.73~4.03)
≧40	12 (63.2%)	7 (36.8%)			3 (50.0%)	3(50.0%)		

p.s. EOT: end of treatment.

**Table 7:** The association between IFN-γ and sustained virologic response in HCV subgroups (RVR vs. non-RVR).

IFN-γ (pg/ml)	RVR				non-RVR			
	SVR	Non-SVR	X2 or Fisher’s	Odds ratio	SVR	Non-SVR	X2 or Fisher’s	Odds ratio
	(n = 55)	(n = 1)	p-value	OR (95% C.I)	(n = 18)	(n = 18)	p-value	OR (95% C.I)
IFN-γ 4wk								
<180	48(100.0%)	0(0.0%)	0.143	1.14 (0.88~1.49)	16 (61.5%)	10 (38.5%)	0.06	3.08 (0.86~11.03)
≧180	7(87.5%)	1(12.5%)			2 (20.0%)	8 (80.0%)		
IFN-γ 12wk								
<120	40 (100.0%)	0 (0.0%)	0.286	1.07 (0.94~1.21)	13 (68.4%)	6 (31.6%)	0.019	2.33 (1.05~5.16)
≧120	15 (93.7%)	1(6.3%)			5 (29.4%)	12 (70.6%)		
IFN-γ EOT								
<40	39 (100.0%)	0 (0.0%)	0.304	1.06 (0.94~1.20)	13 (68.4%)	6 (31.6%)	0.019	2.33 (1.05~5.16)
≧40	16 (94.1%)	1 (5.9%)			5 (29.4%)	12 (70.6%)		

p.s. EOT: end of treatment.
