# Supplementary material for: Elevated on-treatment levels of serum IFN-gamma is associated with treatment failure of peginterferon plus ribavirin therapy for chronic hepatitis C
Source: Sci Rep. 2016 Mar 11;6:22995. doi: 10.1038/srep22995 (PMC4786849; doi:10.1038/srep22995)
Supplement: Supplementary Information [file srep22995-s1.pdf]

Elevated on-treatment levels of serum IFN-gamma is associated with treatment failure of peginterferon plus ribavirin therapy for chronic hepatitis C

Authors:

Ming-Ying Lu, MD[7]; Ching-I Huang, MD[4]; Chia-Yen Dai, MD, PhD[1,2,7]; Shu-Chi Wang, PhD[1,6]; Ming-Yen Hsieh, MD[1]; Meng-Hsuan Hsieh, MD[2,6]; Po-Cheng Liang, MD[1]; Yi-Hung Lin, MD[1]; Nai-Jen Hou, MD[4]; Ming-Lun Yeh, MD[1,2,7]; Chung-Feng Huang, MD[1,2,5]; Zu-Yau Lin, MD[1,2]; Shinn-Cherng Chen, MD, PhD[1,2]; Jee-Fu Huang, MD, PhD[1,2]; Wan-Long Chuang, MD, PhD[1,2]*; Ming-Lung Yu, MD, PhD[1,2,3]*

Affiliations:

[1]Hepatobiliary Division, Department of Internal Medicine and Hepatitis Center, Kaohsiung Medical University Hospital, Kaohsiung, Taiwan.

[2]Faculty of Internal Medicine, College of Medicine, and Graduate Institute of Clinical Medicine, and Center for Infectious Disease and Cancer Research, Kaohsiung Medical University, Kaohsiung, Taiwan.

[3]Institute of Biomedical Sciences, National Sun Yat-Sen University, Kaohsiung, Taiwan.

[4]Department of Internal Medicine, Kaohsiung Municipal Hsiao-Kang Hospital,

Kaohsiung Medical University, Kaohsiung, Taiwan.

[5]Department of Occupational Medicine, Kaohsiung Municipal Ta-Tung Hospital, Kaohsiung Medical University, Kaohsiung, Taiwan.

[6]Department of Preventive Medicine, Kaohsiung Medical University Hospital, Kaohsiung, Taiwan.

[7]Graduate Institute of Medicine, College of Medicine, Kaohsiung Medical University, Kaohsiung, Taiwan

*Corresponding author: Ming-Lung Yu, M.D., Ph.D.

Hepatobiliary Division, Department of Internal Medicine, Kaohsiung Medical University Hospital, Kaohsiung, Taiwan

Tel: +886-7-312-1101 ext. 7475

Fax: +886-7-312-3955

Email: fish6069@gmail.com

Supplementary Table 1. Baseline serum cytokine expression levels between SVR and non-SVR

Cytokine	SVR	non-SVR	p-value
IFN-γ (pg/ml, mean±SE)	102.4±20.7	147.8±50.3	0.355
IL-2 (pg/ml)	52.7±6.2	86.9±13.8	0.017
TNF-α (log pg/ml)	1.66±0.11	1.45±0.20	0.389
IL-4 (pg/ml)	363.1±61.3	649.1±139.1	0.040
IL-5 (pg/ml)	126.7±22.9	146.2±47.2	0.696
IL-6 (log pg/ml)	0.74±0.20	0.31±0.29	0.226
IL-10 (log pg/ml)	0.92±0.17	0.77±0.26	0.674
IL-1β (log pg/ml)	0.86±0.09	1.24±0.28	0.219
IL-8 (log pg/ml)	1.43±0.16	0.99±0.22	0.117
IL-12 (pg/ml)	55.7±20.9	46.2±24.1	0.813

Supplementary Table 2. Serial serum cytokine expression levels between RVR and non-RVR

	RVR	Non-RVR	p-value
IFN-γ (pg/ml, mean±SE)			
baseline	108.1±23.9	116.3±32.8	0.839
2 week	89.2±17.2	160.8±43.8	0.136
4 week	92.6±15.3	124.8±32.1	0.315
IL-2 (pg/ml)			
baseline	57.1±6.8	64.2±10.9	0.583
2 week	84.4±11.7	87.6±11.9	0.857
4 week	102.3±15.3	97.0±24.0	0.846
TNF-α (log pg/ml)			
baseline	1.57±0.12	1.71±0.16	0.472
2 week	1.60±0.11	1.74±0.16	0.47
4 week	1.73±0.14	1.63±0.16	0.635
IL-4 (pg/ml)			
baseline	382.8±72.8	494.9±95.9	0.35
2 week	314.3±44.4	463.6±101.5	0.134
4 week	350.9±42.4	384.4±75.7	0.701
IL-5 (pg/ml)			
baseline	122.8±24.9	144.3±36.1	0.614
2 week	130.3±24.9	231.1±58.7	0.123
4 week	161.4±31.6	147.4±43.6	0.792
IL-6 (log pg/ml)			
baseline	0.73±0.22	0.51±0.26	0.511
2 week	0.74±0.21	0.57±0.24	0.588
4 week	0.64±0.20	0.44±0.23	0.525
IL-10 (log pg/ml)			
baseline	0.98±0.19	0.78±0.22	0.507
2 week	1.04±0.21	1.05±0.22	0.983
4 week	0.92±0.19	0.81±0.21	0.68
IL-1β (log pg/ml)			
baseline	0.94±0.11	0.96±0.17	0.926
2 week	0.81±0.10	0.95±0.15	0.442
4 week	0.99±0.11	0.95±0.14	0.82
IL-8 (log pg/ml)			
baseline	1.34±0.18	1.32±0.22	0.94
2 week	1.28±0.16	1.10±0.17	0.44
4 week	1.32±0.18	0.97±0.17	0.157
IL-12 (pg/ml)			
baseline	64.1±25.6	36.4±15.2	0.429
2 week	60.1±20.8	66.1±26.5	0.858
4 week	28.5±7.3	37.7±20.8	0.626

Supplementary Table 3. The association between IFN-γ and SVR in HCV patients

IFN-γ (pg/ml)	SVR (n=69)	Non-SVR (n=10)	X2 or Fisher's p-value	Multivariate p-value	
				OR (95% C.I)	p-value
IFN-γ 4wk					
＜180	61 (92.4%)	5 (7.6%)	2.2×10^{-3}	9.35 (1.63~53.66)	0.012
≧180	8 (61.5%)	5 (38.5%)			
IFN-γ 12wk					
＜120	51 (94.4%)	3 (5.6%)	9.4×10^{-3}	13.19 (2.50~69.52)	2.4×10^{-3}
≧120	18 (72.0%)	7 (28.0%)			
IFN-γ EOT					
＜40	50 (94.3%)	3 (5.7%)	0.012	6.85 (1.47~32.06)	0.014
≧40	19 (73.1%)	7 (26.9%)			

p.s. (1) HCV-1b patients with non-RVR treated less than 48 weeks PegIFN/RBV therapy were excluded. (2)Adjusted for GPT and viral load. (3) EOT: end of treatment.

Supplementary Figure 1. Area under the serial IFN-γ expression curve during PegIFN/RBV therapy

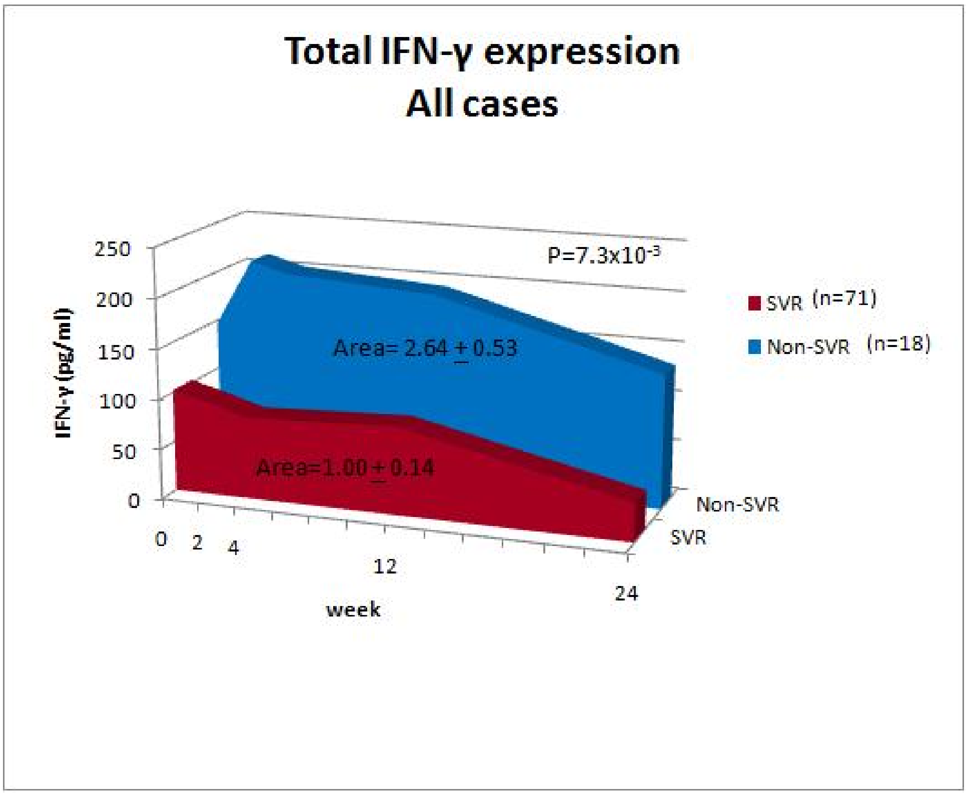

Supplementary Figure 2. The fold change of IFN-γ between SVR and non-SVR

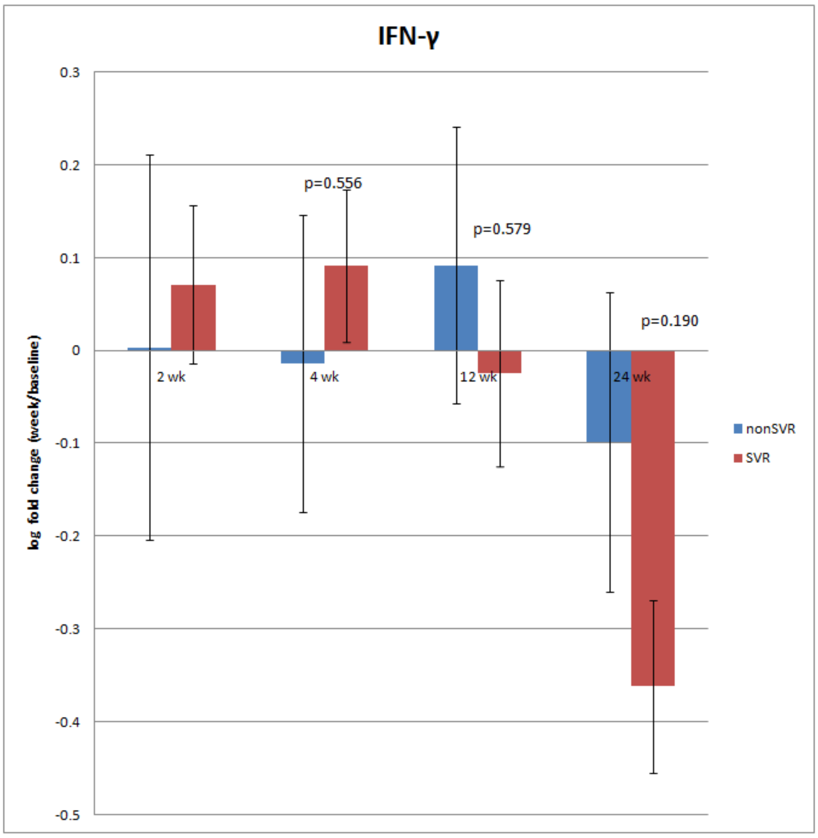

Author contributions

MY.L. and CY.D. designed the study and wrote the main manuscript text. SC.W. performed the experiments and prepared the figures. MY.H., CI.H., MH.H., PC.L., YH.L., NJ.H., ML.Y., CF.H., ZY.L., SC.C., and JF.H. collected the clinical data. ML.Y. and WL.C. designed the study and supervised the statistics. All authors have reviewed the manuscript.
